# Structure-Function Analyses of Human Bitter Taste Receptors—Where Do We Stand?

**DOI:** 10.3390/molecules25194423

**Published:** 2020-09-26

**Authors:** Maik Behrens, Florian Ziegler

**Affiliations:** Leibniz-Institute for Food Systems Biology at the Technical University of Munich, 85354 Freising, Germany; f.ziegler.leibniz-lsb@tum.de

**Keywords:** bitter taste receptor, TAS2R, GPCR, molecular modeling

## Abstract

The finding that bitter taste receptors are expressed in numerous tissues outside the oral cavity and fulfill important roles in metabolic regulation, innate immunity and respiratory control, have made these receptors important targets for drug discovery. Efficient drug discovery depends heavily on detailed knowledge on structure-function-relationships of the target receptors. Unfortunately, experimental structures of bitter taste receptors are still lacking, and hence, the field relies mostly on structures obtained by molecular modeling combined with functional experiments and point mutageneses. The present article summarizes the current knowledge on the structure–function relationships of human bitter taste receptors. Although these receptors are difficult to express in heterologous systems and their homology with other G protein-coupled receptors is very low, detailed information are available at least for some of these receptors.

## 1. Introduction

The human sense of taste is indispensable for the rapid assessment of the chemical composition of food. The concentrations of sodium ions and protons as well as the energy content in the form of carbohydrates and proteins, by means of their building blocks, mono- and disaccharides and L-glutamic acid, respectively, are sensed as salty, sour, sweet and umami. The fifth basic taste quality, bitter, is elicited by a large number of chemically diverse substances of which a considerable proportion is rather poisonous, and hence, their ingestion needs to be avoided [1]. For each of these five basic taste qualities specific receptors or receptor families exist, which are expressed in sensory cells located in the oral cavity [2]. Whereas the sour taste receptor, otopetrin-1 [3,4,5], and the salt taste receptor, ENaC (epithelial sodium channel) [6], represent ion channels, sweet, umami (the taste of L-glutamic acid in humans) and bitter are sensed by G protein-coupled receptors (GPCRs) [7]. The three members of the taste 1 receptor (TAS1R) gene family assemble to form the functional sweet taste receptor heteromer, TAS1R2/TAS1R3 [8,9,10] and the corresponding umami taste receptor, TAS1R1/TAS1R3 [11,12], respectively. The taste 2 receptor (TAS2R) gene family is devoted to the detection of bitter substances [13,14,15].

In humans, the TAS2R gene family consists of ~25 putatively functional members [16]. The number of bitter taste receptor genes is not conserved among vertebrates [17]. Whereas primates and rodents possess similar or slightly higher gene numbers compared to humans, respectively, the gene numbers in other vertebrates can fluctuate between 0 to 3 at the low end and 56 to 74 at the high end [17]. There are initial hints that a low number of functional bitter taste receptors could be partially compensated by elevated average tuning breadths [18]. While the functional profiling of bitter taste receptors in the early years after their discovery was somewhat biased towards human TAS2Rs [16], more recently other vertebrates’ receptors were more avidly investigated. To date, we know at least one bitter agonist for 21 of the 25 human TAS2Rs [16], 21 of 35 mouse Tas2rs [19] and we have such information for several bird species [18], the domestic cat [20,21], rat [22,23], frog [18], fish [24], bat [25] and some primates [26,27,28,29,30,31].

After the successful characterization of about half of the human TAS2Rs and the finding that TAS2Rs can be classified according to the number of agonists into broadly tuned receptors with numerous agonists, narrowly tuned receptors with very few agonists, receptors recognizing selective chemical classes and intermediately tuned receptors [16], the structural features determining the tuning characteristics especially of broadly tuned TAS2Rs moved into the focus of research. However, it was, and still is, a major obstacle that bitter taste receptors only exhibit very minor homology with other GPCR families [32,33] and that experimental structures of TAS2Rs are lacking. Hence, homology modeling was necessary despite the fact that homology was very low. The first attempts to elucidate the structures of TAS2Rs were, therefore, done either by functional experiments combined with mutagenesis of receptor positions believed to play an important role in agonist interaction only [34] or by pure homology modeling and docking experiments [35,36]. The first publication using a combination of functional experiments, point mutageneses and in silico analyses was done with human TAS2R46 [37] and this approach quickly was adopted for subsequent investigations, because it allowed the iterative refinement of the obtained structures. To date, a considerable number of studies have been performed on multiple human and non-human bitter taste receptors, providing insight into the architectures of bitter taste receptor binding sites and how a diverse set of compounds can be accommodated.

Yet, all models published to date still have to be considered low-resolution models [38]. This potential lack in accuracy will likely continue until experimental crystal structures of bitter taste receptors become available. Because the heterologous expression of chemoreceptors, including bitter taste receptors, is considered particularly difficult [14,39,40,41], it is not surprising that these receptors are trailing in the list of successful structure elucidations. Moreover, since bitter taste receptors also share very limited homology among each other, a single crystal structure will likely not fully solve the resolution issues. Another problem that affect the prediction of bitter taste receptor structures and agonist interactions is the difficulty to model extracellular loop (ECL) regions even with low precision [32]. Since the ECL of TAS2Rs are close to the experimentally predicted agonist binding sites, their participation in agonist interactions, although frequently proposed, requires confirmation. This is even more challenging because it was shown that TAS2Rs are glycoproteins exhibiting a highly conserved site for asparagine-linked glycosylation in the center of the second ECL [42], a feature that has not yet been incorporated in homology modeling and docking experiments thus far.

The present article summarizes the current knowledge regarding bitter taste receptor biochemistry and cell biology as well as the methods that are currently applied to investigate their structural features. Then, the localization and architecture of the receptor binding pockets will be detailed before the receptor activation mechanism is discussed. Finally, open questions and future directions are highlighted.

## 2. General Features of TAS2Rs

Although the typical seven transmembrane domains, linked by three intra- and three extracellular loops, an extracellular amino terminus and an intracellular carboxyl terminus clearly earmark the TAS2Rs as members of the GPCR superfamily, the low homology with any other GPCR class (e.g., the amino acid sequence of chicken Tas2r1 shares about 10% identity and 30% similarity at the most with currently crystallized GPCRs [38]) makes the appropriate integration into the superfamily difficult. Whereas some researchers propose a common branch with frizzled-receptors based on few conserved amino acid sequence signatures [33], others favor the grouping with class A/Rhodopsin-like GPCRs because of the similarities shared with respect to the localization and architecture of the ligand-binding pockets [32].

Similar to the large odorant receptor (OR) family, TAS2Rs are difficult to express in heterologous mammalian cell lines. A successful strategy to overcome this problem was to connect the receptors’ coding sequences with N-terminal residues of other GPCRs such as the N-terminal 20 or 39 amino acids of bovine rhodopsin [14,43] (named “rho-tag”) or the N-terminal 45 amino acids of the rat somatostatin receptor subtype 3 [22,44] (named “sst3-tag”) and to use these chimeric receptors for functional heterologous expression assays. These amino terminal sequences have been coined “export-tags” to highlight their roles in the routing of receptors to the plasma membrane. As the necessity to generate fusion proteins for functional expression in vitro indicated that chemosensory cells may possess factors that facilitate functional expression in vivo, which are lacking from heterologous cells, researchers have been searching for these factors. For ORs, specific members of two small gene families of auxilary factors, the receptor transporting proteins (RTP) 1–4 and the receptor expression enhancing proteins (REEP) 1–6, were identified [45]. Later, it turned out that some of the bitter taste receptor subtypes also benefited from the co-expression of distinct members of the RTP family, namely RTP3 and RTP4 [46]. Whereas some TAS2Rs become trapped in intracellular compartments of human embryonal kidney cells when expressed without sst3-tag, RTP3- and likewise RTP4-coexpression resulted in elevated levels of these receptors at the cell surface [46]. Another important feature contributing to the levels of functional TAS2Rs at the cell surface in heterologous cell lines is the existence of an N-linked oligosaccharide moiety in the center of the second ECL, which has been predicted by the presence of a highly conserved consensus sequence in all 25 human TAS2Rs [42]. Similar to the observations of native TAS2Rs without export-tags being insufficiently expressed at the cell surface, TAS2Rs without an oligosaccharide-side chain attached to their second extracellular loop also show functional deficits when expressed in heterologous cells. Intriguingly, co-expression of RTP3 and RTP4 partially restored the function of non-glycosylated TAS2R16, suggesting that ECL2-glycosylation is not required for acute receptor function, but rather for proper folding and/or receptor routing to the plasma membrane [42]. Surprisingly, also the presence of certain agonists may promote cell surface localization of TAS2Rs. This has been shown at the example of quinine, a bitter and membrane-permeable natural compound, and TAS2R4 as well as four additional human TAS2Rs, indicating that quinine may act as pharmacochaperone [47]. Other test substances, including the likewise amphiphilic, membrane-permeable substance dextrometorphan did not show a comparable effect, indicating that this activity of quinine might be rather exceptional.

Similar to numerous other GPCRs (for a review see [48]), TAS2Rs can form oligomers [49]. As the 25 human TAS2Rs co-expressed in vitro have been demonstrated to readily form homomers as well as heteromers with each other, one can assume that the population of bitter taste receptor cells (TRC) in vivo could possess ~325 distinct receptor dimers. Indeed, it was assumed that such a large number of different receptors might be necessary to detect all the diverse bitter substances present in nature. However, it turned out that no functional changes that could be attributed to heteromeric TAS2Rs could be observed. Instead, the existing agonist profiles of TAS2Rs were sufficiently explained by the homomeric receptors [49,50]. In light of the extremely broad tuning of especially TAS2R10 [22], -R14 [51] and -R46 [52], each recognizing about one-third of all bitter substances and combined about half of them [16], heteromerization may not be necessary to broaden the receptive range of the TAS2R repertoire [17]. Not all TAS2Rs are broadly tuned; in fact, several receptors are very narrowly tuned responding only to one–three bitter compounds, whereas the majority of the TAS2Rs exhibit intermediate tuning breadths. At present, four TAS2Rs remain orphan [16,53].

Compared to other GPCRs, TAS2Rs are rather insensitive, detecting their agonists at concentrations between the mid nanomolar and the low millimolar range [54]. As taste receptors in the oral cavity are confronted with concentrated mixtures of chemicals from food items, an elevated sensitivity would not necessarily be beneficial, but could rather lead to the rejection of edible food, which would be an evolutionary disadvantage. It has to be noted though that the expression of bitter taste receptors is not limited to the oral cavity. The list of non-gustatory tissues that possess bitter taste receptors is long and the extraoral expression and function of taste receptors has been subject to several comprehensive reviews recently [55,56,57,58,59,60]. Therefore, it should just be mentioned here that the function of TAS2Rs is not limited to gustation, but extends to roles in innate immunity and respiratory function, regulation of digestive function and metabolism as well as male fertility. Hence, the physiological roles of TAS2Rs clearly go beyond taste and tastant detection, an interaction with endogenous agonists and metabolites must be taken into account.

The canonical signal transduction cascade in human bitter taste receptor cells has been described in much detail in a variety of review articles [2,61,62,63]. Briefly, the activation of TAS2Rs by bitter agonists result in the activation of a heterotrimeric G protein composed of Gα-gustducin, a Gαi-type subunit identified first in the gustatory system [64], Gβ3 (Gβ1) [65] and Gγ13 [66]. Upon dissociation of the activated G protein, the βγ-subunits activate phospholipase Cβ2 (PLCβ2), resulting in the production of the second messenger inositol 1,4,5-trisphosphate (IP_3_) along with diacylgylcerol from the signaling precursor phosphatidylinositol 4,5-bisphosphate (PIP_2_) [67]. Next, IP_3_ binds to its ER-membrane-resident receptor, IP_3_R3, which, upon opening, allows the flux of calcium ions stored in the ER-lumen into the cytoplasm [68,69]. The increased cytosolic calcium level in turn triggers the opening of the transient receptor potential channel TRPM5 [70] in the plasma membrane, leading to an influx of extracellular sodium ions causing the cell to depolarize, which is followed by action potentials generated through voltage-gated sodium channels [71]. Finally, the neurotransmitter adenosine 5′-triphosphate (ATP) [72] is secreted through the calcium homeostasis modulator 1 (CALHM1) [73], a pore-forming voltage-gated channel, and purinergic afferent nerve fibers are activated [72] to transmit the signal to the brainstem.

## 3. Different Approaches to Investigate TAS2Rs

### 3.1. Obtaining Experimental Structures

To assess ligand binding to GPCRs and the existence of intramolecular networks that result in structural transformations involved in receptor activation, experimental structure determination would be extremely valuable. Even though the number of experimentally solved structures by crystallization and X-ray diffraction as well as cryo-EM approaches almost 70 (https://zhanglab.ccmb.med.umich.edu/GPCR-EXP/, accessed August 2020), not a single bitter taste receptor is included. Since TAS2Rs were not considered as relevant drug targets until very recently, one could imagine that these receptors were not ranked as a top priority for such kind of analyses. The findings that some bitter compounds represent highly efficient drugs for asthma treatment [74] may serve as alternative anti-diabetic drugs [75], and could even become relevant for the treatment of cancer [76], may result in a shift of priorities.

Ideally, the necessary material to prepare receptors for structure determination can be accessed from natural tissue sources in which the receptor of interest is strongly expressed at high concentrations. Not surprisingly, early receptors successfully subjected to initial structure determinations or even X-ray crystallography were the nicotinic acetylcholine receptor from the electrical organ of the electric ray Torpedo marmorata [77] and bovine rhodopsin that could be purified from bovine retinas [78], respectively. Unfortunately, chemosensory tissues, such as main olfactory epithelia, epithelia of vomeronasal organs or gustatory papillae, represent perhaps the worst source for such purification attempts, because of their small sizes, mixed cell and receptor populations interspersed with numerous non-sensory cells and with high, lifelong turnover [79,80,81].

To obtain large quantities of chemoreceptors, researchers relied on prokaryotic and eukaryotic overexpression systems. Thus far, prokaryotic overexpression and purification has not convincingly resulted in properly folded and functional chemoreceptors, although some publications reported that bacterially-produced taste receptors specifically interact with their agonists in particular as part of biophysical sensors [82]. However, the rather disappointing outcome of bacterial overexpression of chemoreceptors applies only to full length receptors including the integral membrane-associated 7-TM region. Globular extracellular domains have been successfully produced and used to assess ligand binding characteristic with excellent results [83,84]. Most researchers favor the use of eukaryotic cells ranging from yeast to mammalian cells to overexpress chemoreceptors (for a recent review, see [39]). Yet, as outlined above, a plethora of problems make chemoreceptor expression in eukaryotic cells demanding, and this is likely among the reasons for the absence of experimental structures to date.

### 3.2. Homology Modeling

To get first insights into the structure and function of bitter taste receptors, in silico modeling of the peptide sequences has been performed using two different approaches. A pretty straightforward method relies on the homology of a peptide sequence with another peptide sequence for which a crystal structure exists [36]. It is assumed that sequence similarities translate into structural similarities, and hence, the query sequence is more or less simply folded onto the structure determined for the template sequence. This can be done with the help of commercial software packages or free online tools. Depending on the degree of homology, the initial alignment of the query and template sequences can be very demanding or rather easy if homology is extensive. Unfortunately, in addition to the initial resolution of the structurally analyzed template receptor, a low degree of homology will result in low resolution models. This is certainly the case for bitter taste receptors which exhibit very low sequence similarity with any other GPCR-family [38,85]. Knowing that the homology of TAS2Rs with other GPCRs is low, and thus that homology modeling can only provide low-resolution models, another approach to model TAS2Rs, called ab-initio modeling, was suggested. Here, the entire model was prepared in silico without using a specific template. Only few models were generated using this method [35,86].

After molecular modeling, the generated receptor structure can be used for in silico docking experiments that usually require the suitable preparation of ligand structures, a feature that is included in the above-mentioned software packages. It should be noted that ligand docking into a receptor model typically requires the researcher to decide whether and which receptor residues are designated as “flexible” and where in the receptor model the docking should take place. Ideally, this is done after determining potential binding pockets with sufficient volumes to accommodate the ligand(s), a feature also commonly available in commercial software packages. After docking of cognate ligands, the results ideally correlate with observed experimental data for the modeled receptor on both, the qualitative level as well as the quantitative level, such that only ligands that have been shown to interact with the receptor are docked with high scores and that the experimentally observed rank order of potencies is mirrored by the observed docking scores. Here, another problem with bitter taste receptors has to be mentioned; compared to other GPCRs, bitter taste receptors show low affinity between ligands and receptors [87]. In general, one needs to anticipate rather low docking scores, which may be close to, or overlapping with, alternative docking poses, leaving the determination of the most likely docking pose to the scientist.

### 3.3. Functional Heterologous Expression in Combination with In Vitro Mutagenesis

**Figure 1:**
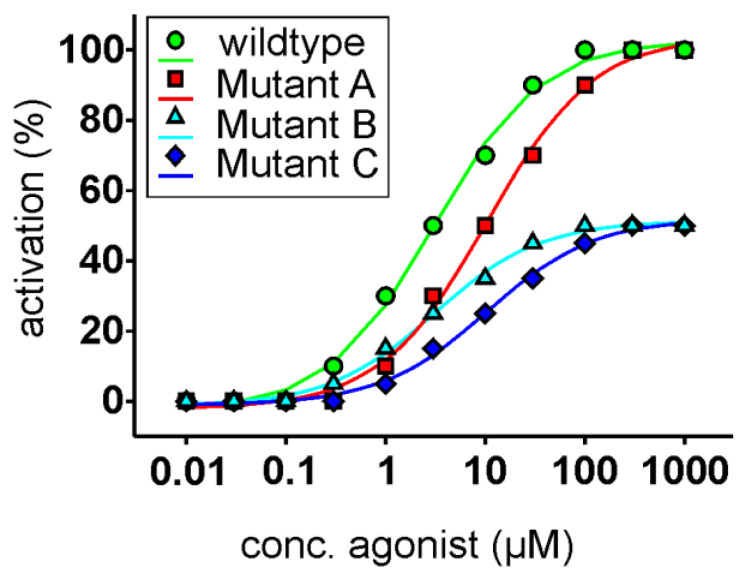
Idealized dose-response relationships of functional receptor assays. The unmodified receptor (circles, green) responses (x-axis, set to 100%) are plotted as a function of the agonist concentration (x-axis, logarithmically scaled). The fictitious receptor mutant A (squares, red) shows a right shift of the curve; however, it reaches the maximal response magnitude of the unmodified receptor. Since only the agonist concentration needs to be raised to achieve an activation similar to the unmodified receptor, the interaction of the agonist with the receptor’s binding pocket seems to be weakened by this mutation. The fictitious receptor mutant B (triangles, cyan) exhibits only maximal amplitudes of 50% of the unmodified receptor; however, the EC_50_-concentration (concentration at which half-maximal receptor activation is reached) is identical. In this case, one can assume that the residue mutated in mutant B is important for the activation by the agonist but not in the binding, since identical agonist concentrations result in similar proportional receptor activities. Finally, fictitious receptor mutant C (diamonds, blue) shows a drop in maximal signal amplitude as well as a shift in the EC_50_-concentration, making interpretations of the underlying reason(s) difficult to impossible. In fact, this type of behavior is sometimes observed when in vitro mutagenesis is combined with functional assays.

Because of a lack of experimental structures and the problems that arise from low resolution homology models, the current gold standard for the determination of receptor structures is composed of a combination of molecular modeling and docking with wet lab experiments, such as functional expression assays and in vitro mutagenesis of the investigated receptors. The combination of these experiments can be performed in an iterative fashion resulting in progressive improvements of in silico models. Again, chemoreceptors, including bitter taste receptors, possess specific problems that arise from their generally low-affinity interactions with their ligands; real experimental binding studies have not been successfully performed, and hence, binding is only assumed as a prerequisite for the commonly-monitored receptor activation. Therefore, wet lab researchers and in turn bioinformaticians using the data to refine their models have to be very cautious when interpreting data arising from functional receptor assays (see Figure 1).

**Figure 2:**
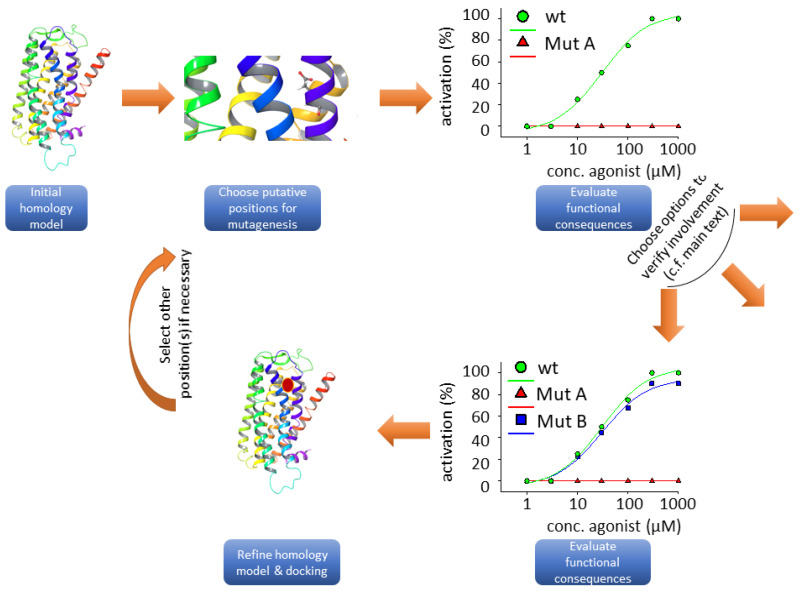
Flow chart illustrating the typical steps involved in structure-function studies. The taste 2 receptor (TAS2R) ribbon model was taken from bitterDB [88,89]. The graphs do not rely on experimental data; they were drawn for illustration purposes only.

In fact, the number of potential effects resulting in functional changes of receptors by far exceeds the few mentioned in the legend of Figure 1. The number of functional receptors at the cell surface can be modified by point mutations, which result in misfolding and mis-routing of the newly synthesized protein. Tentatively, and if properly folded and routed receptors are still formed, one would assume that the magnitude of signaling rather than the threshold or EC_50_-concentrations should change; however, in cases of severe depletion of functional and cell surface associated receptors, shifts in these parameters are conceivable as well. Moreover, a plethora of additional complications (e.g., protein stability, G protein-coupling, desensitization, improper posttranslational modification, etc.), which cannot be discussed here, could be envisaged as underlying causes for functional changes of mutated bitter taste receptors. Hence, astounding and sapid effects caused by receptor mutations can be rapidly generated and, with a low-resolution model at hand, also acceptably illustrated and explained; however, experimental controls are crucial for the reliability of the mapped interactions and, ultimately, the receptor model and the generated docking poses. The finding that a point-mutated receptor exhibits reduced or even a complete loss of responses per se may only serve as a first hint for a possible involvement of the modified residue in agonist interaction. Partial or complete misfolding of the receptor or associated/related problems during biosynthesis and trafficking could also explain this observation. There are some general hints for well performed structure-function studies (see Figure 2).

(1) A very laborious but convincing control has been published by Brockhoff et al., who identified by point mutagenesis and functional experiments all residues in TAS2R46 that contributed to strychnine sensitivity. Next, the identified residues were transferred onto the recipient receptors TAS2R31 and TAS2R43, which share considerable amino acid sequence homologies with TAS2R46 but do not respond to strychnine. Subsequent functional experiments confirmed that strychnine sensitivity was also established in the recipient receptors. Hence, it was not only demonstrated that the lack of specific contact points caused reduced strychnine responsiveness, but that the presence of these residues were required and sufficient for strychnine interaction [37]. However, later, it was realized that not all identified strychnine-contacting positions must necessarily interact simultaneously with the agonist, but that some contacts occur in a so-called vestibular binding pocket, which is only transiently occupied, whereas other contacts are limited to the orthosteric binding site of TAS2R46 [90]. (2) The intense and constant search for bitter agonists of human TAS2Rs has resulted in large arrays of cognate agonists for many TAS2R subtypes, in particular for the receptors with broad agonist spectra. A well-performed structure-function study will not rely on single agonists, but select several agonists, favorably a chemically diverse set of agonists. A wider range of test substances bears the chance that mutations that result in reduced receptor responses for one agonist will not affect responses of other agonists. Hence, full functionality of the receptor mutant is demonstrated with one (set of) agonist(s), whereas selectivity of interactions between the mutated position and another agonist (set) is demonstrated. In fact, at least the broadly tuned bitter taste receptors possess binding sites, which are tailored to accommodate multiple diverse bitter compounds at the expense of potentially higher sensitivities for individual agonists by providing different contact points, a feature discovered at the example of the TAS2R10 [91], which exhibited strongly improved responses for some of its agonists caused by point-mutations. Recently, a comprehensive structure-function study performed with the most broadly tuned human TAS2R, the TAS2R14, identified that almost all receptor positions that contribute to the ligand binding site of this receptor, exhibited agonist-selective effects [92]. As some of these positions were considered highly conserved among the TAS2R-family, a drop in agonist activation by point-mutating these positions have been seen with caution, because potential misfolding was suspected. The full functionality of TAS2R14 mutations at these conserved positions with some agonists suggests that misfolding may not be the most likely reason for reduced responsiveness in other receptors as well, and hence, an involvement in agonist interaction appears more likely. (3) Typically, the observation that a mutated receptor position might be crucial for agonist interaction will be the starting point for additional mutations, introducing more subtle changes of the residue in the position of interest. These could also contribute to the reliability of the assumed contact points with the agonist, e.g., if response properties are not negatively affected. (4) If functionality of the receptor cannot be confirmed by one of the above-mentioned tests, different methods to assess proper expression and/or cell surface localization of the investigated receptor mutants should be performed. If an initial receptor model has been generated to, e.g., guide mutagenesis, it is imperative that the model is adjusted in the end to incorporate all knowledge gained from the functional experiments.

## 4. Localization of TAS2R Binding Pockets

As mentioned already, bitter taste receptors are difficult to categorize due to their low amino acid sequence homology with other GPCR families. The ligand binding pocket of class A GPCRs is located at the extracellular side of TM III, V, VI and VII [32]. Indeed, the majority of structure-function studies with bitter taste receptors confirmed the contribution of these TMs in the formation of the ligand binding pocket, although exceptions exist. This may not be too surprising since TAS2Rs are not only rather distantly related to other GPCR-families, but also considerably differ among each other.

In light of the extraordinary broad tuning of the bitter taste receptors TAS2R10, -R14 and -R46, a valid question is if these receptors possess only a single or multiple ligand binding sites to accommodate all the various agonists arose. To address this question, Brockhoff et al. took advantage of the existence of a primate-specific subfamily of eight TAS2Rs, which share vast amino acid sequence homologies but exert very different agonist profiles [37]. The analyses of the residues involved in strychnine responsiveness of TAS2R46, one member of this subfamily, revealed multiple positions affecting the binding of strychnine. The transfer of the identified residues onto the same positions of two other subfamily members, TAS2R31 and TAS2R43, which did not show strychnine sensitivity, resulted not only in the transfer of strychnine responsiveness onto the recipient receptors, but in the transfer of the entire (tested) agonist profile of TAS2R46 [37]. The finding that the bitter taste receptor antagonist 4-(2,2,3-trimethylcyclopentyl)butanoic acid (GIV3727), which inhibits TAS2R31 and TAS2R43 via the interaction with one of the same receptor positions, could interact and inhibit the TAS2R46 modified in a single position confirmed the existence of a single binding pocket [93]. In fact, the binding of such structurally diverse compounds is enabled by the involvement of different subsets of residues within the binding pocket with individual agonists. For the three most broadly tuned receptors, TAS2R10, -R14 and -R46, an involvement of residues in TM III, V, VI and VII has been experimentally confirmed [37,91,92,94,95,96], which is in perfect agreement with the localization of the ligand binding pocket of class A GPCRs. In addition, for TAS2R14 [92] and TAS2R46 [37], the involvement of TM II in agonist binding has been suggested, a fact that might be attributed to the rather spacious shape of the pocket as shown for the TAS2R14 [97]. A modeling and docking study done without in vitro mutagenesis localized the contact points of the antagonist enterodiol in TM III, IV, ECL2, V, VI and VII of the TAS2R10, which is in pretty good agreement with the study published by Born et al. [91]; however, residues in TM IV and ECL2 were exclusively proposed to be involved in enterodiol binding [98]. Whether this could provide a hint on more general differences in the binding of agonists and antagonists to this receptor remains to be determined. Among the more recently discovered agonists of the receptor TAS2R7 are bitter salts [99,100]. Using point mutageneses the contact points for this rather unusual type of stimuli were also mapped to TM III and TM VII [100], which is somewhat different from the study by Liu et al., who investigated the activation of the more conventional organic compound agonists of this receptor and reported instead that residues in TM III, TM V and ECL2 mainly contribute to ligand binding [101]. Compared to most other TAS2Rs, agonist binding to TAS2R16 tentatively should be less complex, because this receptor exhibits a strong bias for structurally similar βD-glucopyranosides [22]. However, it turned out that several structure-function studies showed discrepancies with regard to the binding modes of agonists and, consequently, the receptor positions involved in agonist binding [102,103,104,105]. The most recent report by Fierro et al. concluded that, depending on the agonist, residues in TM II, III, V, VI and VII are responsible for ligand binding [106]. For the human TAS2R38, TM III, V and VI are proposed to be involved in the binding of its agonists phenylthiocarbamide (PTC) and 6-n-propyl-thiouracil (PROP) [107,108,109]. For the TAS2R1, in three autonomous studies, different ligand binding pockets are suggested. Whereas Upadhyaya et al. incorporated experimental mutagenesis studies and assumed TM I, II, III and VI to be involved in ligand binding [110] and Stoeger et al. suggested positions in TM III, TM VI and ECL2 as contact points for L-arginine [111], Dai et al. proposed the putative binding pocket within TM III, V, VI, VII and the extracellular loop 2 (ECL2) [112]. Combining all results of the latest investigations of ligand binding pockets of the human TAS2Rs, TM I and IV seem to be the only transmembrane domains rarely participating in ligand binding. As was already mentioned for the human TAS2R1, besides the transmembrane domains, also the extracellular loops (ECL) are suggested to be involved in ligand binding [34,113]. In TAS2R14, the amino acid residue Arg160, which is located in ECL2, is proposed to participate in the binding of the agonist aristolochic acid [114]. Furthermore, the exchange of ECL1 between TAS2R43 and TAS2R31 leads to the loss of receptor response for TAS2R43 and the gain of responsiveness for TAS2R31 triggered by n-isopropyl-2-methyl-5-nitrobenzenesulfonamide (IMNB) [87].

Besides the orthosteric binding site, for some class A GPCRs, and recently for TAS2R46, the existence of a second vestibular binding site, which is involved in ligand selectivity, is proposed [90]. This vestibular binding site is located at the extracellular part of the receptor and only transiently occupied by agonists. To manage the high amount of different and highly concentrated bitter compounds, this binding site may function as filter for the orthosteric binding site. As extracellular loops are part of this vestibular binding site, these findings are in good agreement with the suggested involvement of extracellular loops in agonist selectivity. For an overview of receptor positions implicated in ligand binding, see Table 1.

## 5. Receptor Activation

The activation mechanism of bitter taste receptors is a poorly investigated challenge. However, most of the conserved motifs of class A GPCRs were shown to have a corresponding counterpart in bitter taste receptors. An N^1.50^xxI^1.53^ motif replaces the N^1.50^xxV^1.53^ motif in TM I and the N^7.49^P^7.50^xxY^7.53^ in TM VII is changed to H^7.49^S^7.50^xxL^7.53^ [32]. For GPCRs in general, conformational changes by ligand binding to the inactive receptor are thought to trigger a receptor response [116]. It is assumed that the conserved motifs may be involved in stabilization of this inactive conformation until agonist binding, as the mutations of isoleucine at position 1.53 in TAS2R1 and serine at position 7.50 in TAS2R4 to alanine result in hyperactive receptors [115,117,118].

Further important amino acid residues for receptor activation were suggested for the human TAS2R38 in analogy to rhodopsin [107]. By X-ray crystallography, two very different structural arrangements in the G protein-bound and unbound state of the bovine rhodopsin were identified [119]. By forming a hydrophobic interaction, the two amino acid residues at positions 6.43 and 7.52 were proposed to be responsible for these structural rearrangements [119]. Transferring this knowledge on the related TAS2R38 suggests an involvement of the amino acids F255 and V296 in receptor activation [107]. By introducing a double mutant, which maintains the putative interaction between F255 and V296, Biarnés et al. were able to show an TAS2R38 response to PTC, which is comparable to the wildtype receptor. These results indicate a critical role of F255 and V296 in TAS2R38 activation [107].

Molecular modeling studies showed the involvement of inter- and intrahelical H bonds in TAS2R1 activation. The amino acid residue N24^1.50^ was shown to establish an H bond network connecting TM I, TM II and TM VII, which is only present in the agonist bound state and absent in the unbound TAS2R1 [115]. Dai et al. further proposed a control switch between the intracellular loop 2 (ICL2) and the cytoplasmic end of TM III in the human TAS2R1. Upon ligand binding, a combination of the opening of this switch and the formation of a helix in the ICL2 are assumed to be involved in TAS2R1 activation. The conservation of amino acid residues involved in the control switch in TAS2Rs suggests a conserved bitter taste receptor activation mechanism [112]. These molecular modeling studies give first insights in putative activation mechanisms of human bitter taste receptors, but experimental evidence is still scarce.

As already mentioned, mutations of amino acid residues of broadly tuned bitter taste receptors have agonist-specific effects [37,91,92]. Most of the identified residues in the human TAS2R14 impaired the potency, as well as the efficacy, of the investigated agonists, but a drop in sensitivity is sometimes correlated with an increase in signal amplitude. These results indicate that amino acid residues, which are involved in ligand binding, may have an additional function in receptor activation [92].

## 6. Outlook

Despite the numerous issues associated with structure–function analyses of bitter taste receptors, research has made considerable progress towards a better understanding of the receptors’ interactions with their various agonists in particular for the broadly tuned generalist receptors. At present, the field is urgently awaiting the availability of experimental models to compare the existing models with experimental data and to facilitate the transition from low to high resolution models, although the authors anticipate that low resolution models supported by vigorous experimental confirmation would likely also allow the de novo prediction of novel agonists and antagonists as well as a better understanding of the receptors’ activation mechanism in the future. However, it will certainly speed up the process if these endeavors could be started with experimental structures in hand. As TAS2Rs are not only very distantly related to other GPCRs, but also among each other, a single crystal structure may not be sufficient to serve as template for the high-resolution modeling of all 25 TAS2Rs. As TAS2Rs, in light of their expression in multiple extraoral tissues and with presumed roles numerous important physiological processes, are considered important drug targets, the development of small molecules for their activity modulation will soon become a very active research field benefiting from structure–function research.

## Figures and Tables

**Table 1:** Positions involved in TAS2R ligand binding. Amino acid residues involved in agonist binding of human TAS2R1, -4, -7, -10, -14, -16, -20, -40 and -46 are presented. Positions are indicated according to Ballesteros-Weinstein numbering (BW pos.). The interacting amino acid, its position and the corresponding references are displayed.

BW Pos.	TAS2R									
	1	4	7	10	14	16	20	38	40	46
2.53						L59 [105 #, 106 *]				
2.57						S63 [106 *]				
2.60			D65 [101 #]				H65 [95 #]			N65 [90]
2.61	N66 [110 *, 115]				W66 [92,94 *]	N67 [106 *]				W66 [37, 90, 96 *]
2.65										E70 [37, 90, 96 *]
2.66										L71 [37, 90, 96 *] a
ECL1	E74 [110 *, 115] b						T74 [95]			
3.24						V77 [105 #]				
3.25		S81 [95]								I82 [37, 90, 96 *] c
3.28					L85 [92]	L81 [105 #]				
3.29	C82 [111 *]		D86 [101 #]	S85 [91, 96 *]	T86 [92, 95 #]	T82 [105 #]			K98 [94 *]	Y85 [90]
3.30					N87 [92]					N86 [90]
3.32	L85 [94 *]		W89 [101 #]	W88 [91]	W89 [92, 114 *]	W85 [106 *]	W88 [95 #]		T101 [94 *]	W88 [37, 90, 96 *]
3.33	L86 [94 *, 112 *]			V89 [91, 96 *]	T90 [92]	E86 [103, 106 *]				A89 [90]
3.35						F88 [106 *]				
3.36	N89 [94 *, 110 *, 115 *d]			N92 [91, 96 *]	N93 [92, 94 *]	N89 [106 *]		N103 [107, 108]	N105 [94 *]	N92 [37, 90, 96 *]
3.37	E90 [110 *, 112 *]		H94 [100]	Q93 [96 *]	H94 [92]	I90 [105 #]				H93 [90]
3.39						T92 [106 *]				
3.40						F93 [103 #, 106 *]				N96 [90]
3.41						W94 [103 #]		W108 [109 *#]		
3.42								L109 [109 *#]		
3.45								C112 [109 *#]		
4.60	I140 [94 *]								F156 [94 *]	I147 [90]
4.62						S144 [105 #]				
4.64	H144 [94 *]				I148 [94 *, 95 #]					
4.65										N150 [37, 96 *]
ECL2						N148 [105]				
ECL2		Q152 [95]								
ECL2		S154 [95]								
ECL2					R160 [95]					
ECL2					R161 [95]					N161 [96 *]
ECL2	N163 [110 *]	R163 [95]			K163 [95]					
ECL2	A164 [111 *]						W164 [95]			
ECL2		N165 [95]								
ECL2		T166 [95]					I166 [95]			
ECL2			N167 [101]							
ECL2	K168 [112 *]									
ECL2			T169 [101]							
ECL2			W170 [101]							
5.38			S181 [101 #]		L178 [94 *]					S175 [90]
5.39	Q175 [94 *]			K174 [96 *]	I179 [95 #]	Q177 [103 #]			L194 [94 *]	N176 [37, 90, 96 *]
5.40				Q175 [91, 96 *]						
5.42	S178 [94 *]			L177 [96 *]	T182 [92, 95 #]			F197 [108]		V179 [96 *]
5.43				L178 [91, 96 *]	S183 [92]	H181 [103 #]				T180 [90]
5.44								Y199 [109 *#]		
5.46	E182 [94 *, 112 *]				F186 [92]	A184 [105 #]		W201 [108]		
5.47					I187 [92]					N184 [90]
5.48								V203 [109 *#]		
5.49								P204 [109 *#]		
6.48	Y237 [112 *]				Y240 [92]	F236 [106 *]				
6.49					A241 [92]					
6.51				Y239 [91]	F243 [92, 94 *]	Y239 [106 *]				Y241 [37, 90, 96 *]
6.52						F240 [103 #]		S260 [108]		F242 [90]
6.54	I243 [94 *]							A262 [109 *#]		S244 [90]
6.55	K244 [94 *, 112 *]				F247 [92, 94 *]	I243 [103#]		A263 [109 *#]	L263 [44 *]	I245 [90]
6.56								F264 [108]		
6.57								I165 [109 *#]		
6.58		Q249 [95]						S266 [109 *#]		S248 [90]
6.59			T255 [101 #]		V251 [95 #]		F249 [95#]			V249 [90]
6.60	S248 [111 *]									
6.63										E253 [37, 96 *]
7.32			E264 [100]							
7.35	I258 [94 *]	K262 [95]			I262 [94 *]	L258 [105 #]				F261 [90]
7.36					I263 [92]					
7.38	F261 [94 *]					W261 [105 #]				
7.39	F262 [94 *]		E271 [101 #]	M263 [96 *]	Q266 [92]	E262 [105 #, 106 *]	Q265 [95 #]		K282 [94 *]	E265 [37, 90, 96 *]
7.42				T266 [96 *]	G269 [92]	V265 [106 *]				A268 [37, 90, 96 *]
7.43						Y266 [106 *]				F269 [37, 90, 96 *]
7.45						F268 [106 *]				
7.46						I269 [106 *]				

(a) assigned ECL1 in [90], (b) assigned BW 2.73 in [115], (c) assigned BW 3.26 in [90], (d) assigned BW 3.45 in [110,115]. * no experimental validation provided in reference, # lacking BW numbering supplemented according to gpcrdb.org.
